# Optimization of *Pickering* Emulsions Containing Andiroba Oil (*Carapa guianensis* Aubl): Physicochemical Characterization, Experimental Approach, and Rheological Behavior for Topical Use

**DOI:** 10.1021/acsomega.5c07298

**Published:** 2025-12-03

**Authors:** Taynara R. A. Xavier, Maria F. C Dias, Kamila L Correa, Ana C. G. A. Freitas, Tais V. G Alves, Camilo B. Teixeira, Antônio Rodrigues, Roseane M. Ribeiro-Costa, José Otávio C Silva

**Affiliations:** † Pharmaceutical and Cosmetic R&D Laboratory, Faculty of Pharmacy, Federal University of Pará, Belém 66075-900, Brazil; ‡ Faculty of Food Technology and Engineering, Institute of Technology, Federal University of Pará, Belém 66075-900, Brazil; § Pharmaceutical Nanotechnology Laboratory, Faculty of Pharmacy, Federal University of Pará, Belém 66075-900, Brazil

## Abstract

Amazonian vegetable oils, due to their medicinal properties, have become essential raw materials in the development of pharmaceutical and cosmetic products, with andiroba oil (*Carapa guianensis* Aubl.) standing out. Classical emulsions, which use conventional emulsifiers, often present stability limitations. In this context, *Pickering* emulsions stabilized by solid particles emerge as a promising alternative. This study aimed to characterize and optimize *Pickering* emulsions containing andiroba oil and magnesium aluminum silicate (Veegum). The physicochemical characterization of the oil was performed according to the guidelines of the *American Oil Chemists’ Society*. X-ray diffraction (XRD), size, polydispersity index (PDI) and zeta potential (ZP) analyses of Veegum were performed. For optimization, a Rotational Central Composite Design (RCCD) was used, with analysis of variance (ANOVA) to evaluate the significance of the variables. The results showed that the oil had an acidity index of 8.90 ± 0.13 mequiv KOH·g^–1^ and a saponification index of 194 mg KOH/g, indicating the presence of beneficial fatty acids. Fourier transform infrared (FT-IR) analyses confirmed characteristic functional groups. Regarding the XRD of Veegum, peaks characteristic of clay was found, with emphasis on 19.45; 21.68; 25.27°, suggestive of the interlamellar space of the material. For the particle size, a value equal to 1299 nm ± 0.04 was obtained with a PDI of 0.672 ± 0.3, suggestive of aggregation and with a ZP of −38 mV ± 0.02, consistent with the silanol groups of Veegum, which favors the formation of stable dispersions. Out of the 19 initial formulations, three were selected, with emulsion 22 standing out due to its ZP of −40.5 ± 0.8 mV, creaming index of 0%, and pH of 7.45 ± 0.72, suitable for topical use. Rheological analysis revealed pseudoplastic behavior, fitting the Herschel–Bulkley model. It was concluded that experimental design is a valuable tool for the development of stable emulsions, and formulation 22 proved to be promising for topical applications.

## Graphical abstract

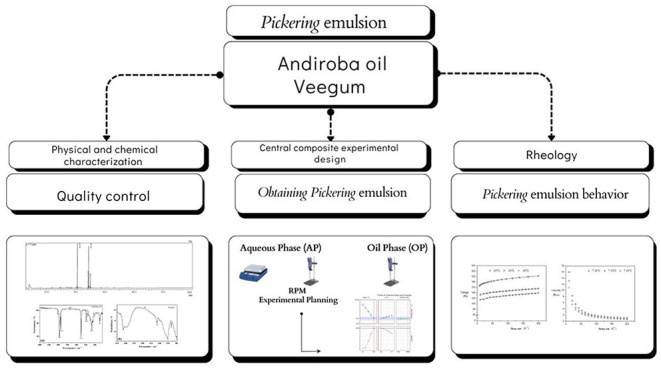

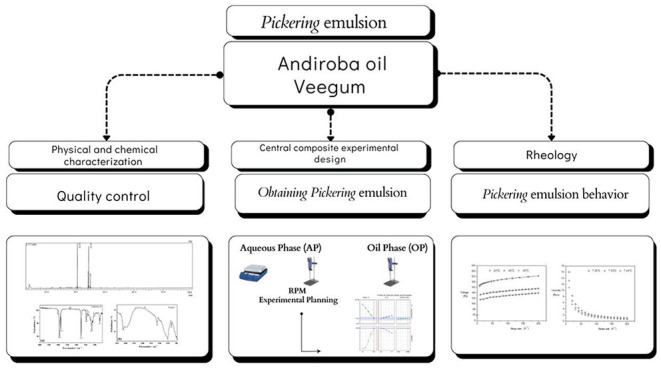

## 1 Introduction

Vegetable oils, especially those derived from the Amazonian biodiversity, have garnered increasing interest in the pharmaceutical and cosmetic industries due to their intrinsic medicinal properties. These oils, rich in bioactive compounds such as unsaturated fatty acids and natural antioxidants, offer a wide range of therapeutic applications.^1^ Among them, andiroba oil (AO), extracted from the seed of the andiroba tree (*Carapa guianensis* Aublet), stands out for its remarkable anti-inflammatory and wound-healing activities, positioning it as a promising raw material for various topical applications.^2^ However, its lipophilic nature, coupled with its susceptibility to oxidation and phase separation in classical emulsions, represents a significant challenge for the efficacy of products using this oil as an active ingredient in their formulations, thus limiting its use in the pharmaceutical industry.^3^

Classical emulsions, which rely on chemical emulsifiers for stabilization, often face problems such as surfactant degradation and formulation instability over time. This is because these conventional emulsions are heterogeneous liquid dispersion systems in which one liquid phase is distributed in the other, stabilized by surfactants or amphiphilic polymers that reduce interfacial tension, creating an electrostatic repulsion or steric barrier between the droplets.^4^ However, these emulsions are thermodynamically unstable and prone to film rupture, in addition to presenting environmental problems associated with the use of surfactants, such as water pollution and low degradability. Given this scenario, there is a need to develop innovative systems, such as *Pickering* emulsions, which contribute to the stability and therapeutic efficacy of andiroba oil in topical formulations.^5^

*Pickering* emulsions are notable for their ability to use solid particles, such as aluminum magnesium silicate, a clay commercially known as Veegum, to stabilize emulsions. This approach is particularly advantageous for formulating andiroba oil, as the addition of Veegum promotes the formation of a colloidal network that improves emulsion stability and prevents phase separation. Furthermore, the solid particles act as barriers that control the release of active compounds, enhancing the bioavailability of the active principles contained in andiroba oil. This controlled release not only increases therapeutic efficacy but also contributes to a more consistent and long-lasting topical application experience.^6^ In contrast to conventional emulsions, *Pickering* emulsions exhibit better adhesion tolerance, biological and environmental compatibility, and are less susceptible to instability and skin irritability. This, along with their low toxicity, opens new possibilities for applications in various fields, including the pharmaceutical and cosmetic industries.^7^

However, developing a stable *Pickering* emulsion with andiroba oil is not trivial. The optimal ratio between components (oil, solid particle, and aqueous phase) and processing parameters (such as stirring speed and time) are critical to the success of the formulation. Inadequate selection of these variables can result in unstable emulsions with rapid phase separation, creaming, or flocculation, invalidating their practical application. Thus, this work aims to solve a formulation optimization problem: to identify the ideal experimental conditions to produce an andiroba *Pickering* emulsion with maximum physicochemical stability.

*Pickering* emulsions represent an innovative approach to emulsion stabilization, and optimization is crucial to maximize their effectiveness and stability. For this, the application of experimental design methodologies, such as Rotational Central Composite Design (RCCD), proves extremely valuable. This robust approach allows for the simultaneous evaluation of multiple factors and their interactions, enabling the identification of ideal conditions that maximize both the stability and effectiveness of the emulsion. This is confirmed through desirability, which provides predictive responses indicating a possible best formulation based on the elective criterion of the study’s hypothesis, which would be to develop the best production conditions. Furthermore, the use of RCCD can optimize product development time and costs, which is especially relevant in industries.^8^

It is important to emphasize that the scope of this study is directed toward the development and optimization of the physicochemical stability of *Pickering* emulsions through the analysis of dependent variables whose responses are essential, such as Zeta potential, creaming index, and pH. Although the developed formulation has potential topical application, the evaluation of functional properties, such as antimicrobial activity, in vivo wound-healing efficacy, or complete toxicological studies, falls outside the objective of this development stage, representing a limitation of the present work and a perspective for future studies.

Therefore, the objective of this study was to develop and optimize *Pickering* emulsions containing *C. guianensis* Aublet oil and aluminum magnesium silicate (Veegum), aiming to define an emulsion with the best desirability characteristics to promote future steps such as stability determination and drug incorporation.

## 2 Experimental Section

### 2.1 Materials

Andiroba oil (*C. guianensis* Aublet) was supplied by *Amazon Oil Industry* (Pará, Brazil) and extracted using cold pressing and filtration techniques, stored at room temperature (20–25 °C). Magnesium aluminum silicate (Veegum) was purchased from Mapric (São Paulo, Brazil) and stored at 15–30 °C.

The following reagents and their respective analytical grades were used: glacial acetic acid (Synth, analytical grade), 37% hydrochloric acid (HCl, Sigma-Aldrich, São Paulo, Brazil), ultrapure water was obtained using a Milli-Q purification system (Merck), sodium benzoate (CRQ Chemicals, São Paulo, Brazil), potassium bromide (KBr, Sigma-Aldrich, São Paulo, Brazil), chloroform (Synth, analytical grade, São Paulo, Brazil), potassium iodide (KI, Synth, analytical grade, São Paulo, Brazil), ethanol (Synth, analytical grade), *n*-hexane (Synth, analytical grade, São Paulo, Brazil), potassium hydroxide ≥ 85% (KOH, Sigma-Aldrich, São Paulo, Brazil), methanol 99.8% (MeOH, Synth, São Paulo, Brazil), 1% aqueous starch solution (Synth, São Paulo, Brazil), Hanus solution (IBr in acetic acid, Sigma-Aldrich, São Paulo, Brazil), 1% ethanolic phenolphthalein solution (Synth, analytical grade, São Paulo, Brazil), and sodium thiosulfate (Na_2_S_2_O_3_, Synth, analytical grade, São Paulo, Brasil).

### 2.2 Physicochemical Characterization of Andiroba Oil

Andiroba oil underwent physicochemical characterization using methods established by the American Oil Chemists’ Society (AOCS). The acid value, peroxide value, and refractive index at 40 °C were evaluated according to protocols AOCS Cd 3d-63, Cd 8b-90, and Cc 7–25, respectively, while the saponification value and iodine value were determined using recommended practices Cd 1c-85 and Cd 3a-94, respectively. The oxidative stability index (OSI) was assessed using a Rancimat apparatus (Metrohm 743, Herisau, Switzerland) at 110 °C, under an air flow of 20 L/h, using 5 g of oil, following the AOCS Cd 12b-92 method.^9^ The oil viscosity was measured using a capillary viscometer (model Ct52, Schott-Geräte GmbH, Mainz, Germany) at a temperature of 40 °C.^9^ Experimental analyses were performed in triplicate, and the results were expressed as means ± standard deviations.

#### 2.2.1 Fatty Acid Profile Determination of Andiroba Oil

For the characterization of the fatty acid profile of andiroba oil, gas chromatography coupled to mass spectrometry (GC-MS, model 6890/5973 V, Shimadzu, Japan) was employed, using an SH-Rtx-5 capillary column (30 m × 0.25 mm) and helium as the carrier gas (1.5 mL/min). Initially, the triglycerides were converted into fatty acid methyl esters through a saponification reaction with KOH/MeOH (0.1 M), followed by esterification with HCl/MeOH (0.12 M). The analyses were performed under the following operational conditions: injection volume of 1 μL in split mode (1:50), with the injector and detector maintained at 250 °C. The column temperature program started at 60 °C (2 min), heated at 10 °C/min to 200 °C, followed by an increase at 2 °C/min to 240 °C; this temperature was held for 24 min. The identification of the compounds was performed by comparing the obtained mass spectra with the standards from the NIST database (Shimadzu, Kyoto, Japan).^10^

#### 2.2.2 Physicochemical Characterization of Andiroba Oil and Veegum: Fourier Transform Infrared Spectroscopy (FT-IR) and Thermogravimetric Analysis (TGA)

Infrared spectroscopy analyses were performed on an FT-IR spectrometer (IR Prestige-21, Shimadzu, Kyoto, Japan) with 32 scans. For the oil, the attenuated total reflectance (ATR) method with a diamond crystal cell was used. For Veegum, the pressed pellet technique with potassium bromide (KBr) was employed. All analyses were conducted at room temperature, in the spectral range of 4000 to 400 cm^–1^.^11^

Furthermore, andiroba oil and Veegum were also subjected to thermogravimetric analysis (TGA) using a TGA analyzer (model 50H, Shimadzu, Kyoto, Japan) under a nitrogen atmosphere with a flow rate of 50 mL/min, a heating rate of 10 °C/min, in a temperature range from 25 to 600 °C, using 9 mg of each sample in aluminum crucibles.^12^

### 2.3 X-Ray Diffraction (XRD) of Veegum

The analyses were performed on a D8 Advance diffractometer, Bruker (Billerica, USA), with a Cu tube, Cu radiation (Kα1 = 1.540598 Å), angular range (°2θ) = 10–80°, tube voltage = 40 kV, tube current = 40 mA, divergent slit = 0.6 mm, Soller slit = 2.5°, Ni Kβ filter. The diffractograms were collected with an angular step of 0.02° and a time per step of 1 s.^13^

### 2.4 Zeta Potential, Average Particle Size (Z-Average), and Polydispersity Index (PDI) of Veegum

For the determination of the Polydispersity Index (PDI) and the average particle size, the samples were diluted at 5:1000 (w/w) in ultrapure water and analyzed at 25 °C. The analysis was performed by dynamic light scattering (DLS) using a Zetasizer Nano-ZS90 instrument (Malvern Instruments Ltd., Worcestershire, U.K.), with a standard quartz cuvette (model ZEN0042).^14^

For the determination of the surface charge (Zeta Potential), the samples were prepared at the same dilution ratio (5:1000 in ultrapure water) and analyzed by the electrophoretic mobility method using the same equipment, with a dedicated Zeta potential cell (model DTS1070) and ultrapure water as the standard. The Zeta Potential results were expressed in mV, and all determinations were performed in triplicate.^14^

### 2.5 Obtaining and Optimization of *Pickering* Emulsions

The emulsions were prepared by combining distilled water, andiroba oil, and aluminum magnesium silicate powder (Veegum). The quantities of reagents and stirring conditions were determined based on an experimental design study, as detailed in Table 4. The preparation process was divided into two phases: the aqueous phase (AP) and the oil phase (OP). In the AP, Veegum is dispersed in water. For this, Veegum is weighed in a beaker, followed by the addition of distilled water and sodium benzoate (0.5% w/v) as a preservative, according to the adapted methodology.^15^ All components of the AP were stirred on a magnetic stirrer (IKA C-MAG HS hot plate stirrers, Campinas, São Paulo) at 300 rpm for 5 min. Subsequently, the AP underwent a more intensive dispersion process using an UltraTurrax T25 digital homogenizer (IKA, Campinas, São Paulo).^16^

The second phase involves emulsifying the two phases. The OP consists solely of andiroba oil (AO); after weighing, the OP was poured into the AP. Then, the mixture was homogenized using an UltraTurrax T25 digital device (IKA, Campinas, São Paulo) at a speed of 22,000 rpm for 5 min. After this process, the samples were left to rest for 24 h before being packaged.^17^

#### 2.5.1 Experimental Design Study

A Rotational Central Composite Design (RCCD) (2^3^) with five replicates at the center point was employed, in conjunction with Response Surface Methodology (RSM). The independent variables considered were the concentration of Veegum (% w/w), the concentration of andiroba oil (% w/w), and the stirring intensity of the Ultraturrax, totaling 100 mL for each emulsion. Each independent variable was evaluated at five equidistant levels, coded as −α (minimum level), −1 (low level), 0 (center point), +1 (high level), and +α (maximum level). The selected dependent variables were pH, Zeta Potential, and creaming index.

A total of 19 formulations were obtained (Table 4) from the experimental design, which were analyzed using analysis of variance (ANOVA) with a significance level of 5% (*p* < 0.05), employing the software Statistica 8.0 (Stat Soft Inc., Tulsa, Oklahoma, USA).^18^

#### 2.5.2 Reparameterization and Predictive Model Selection

After the initial adjustment of the quadratic models using the Student’s *t* test, a statistical reassessment of the generated models for the response variables Zeta potential, pH, and creaming index was conducted. We adopted the criterion of the highest adjusted coefficient of determination (adjusted R^2^) as the decision parameter. This indicator not only considers the degree of correlation between predicted and observed values but also penalizes excessive model complexity, providing a more reliable measure of real predictive capability.

To achieve this, parameters with the highest p-values (Student’s *t* test) that were not significant were sequentially removed until the models for each response variable achieved the highest adjusted *R* ^2^. After reparameterization, the models were evaluated using ANOVA. This approach, referred to here as *reparameterization for statistical performance*, prioritized predictive robustness over the isolated significance of terms.^19^

#### 2.5.3 Optimization by the Desirability Function Method

To identify the ideal *Pickering* emulsion formulation, the multivariate optimization methodology using the desirability function proposed by^20^ was employed. This approach allowed the individual optimization of selected response variables (zeta potential, creaming index, and pH) by transforming each into an individual desirability function ranging between 0 (undesirable) and 1 (optimal). The desirability functions were constructed according to the objectives for Zeta potential: maximization in absolute value (aiming for more negative values indicating greater electrostatic stability); Creaming index: minimization (the lower the value, the greater the physical stability); and pH: maintenance within the optimal range for topical applications (6.0–7.5), with maximum desirability considered at the central value. The analysis was performed using *Statistica 8*.*0 software*.

##### 2.5.3.1 pH Determination

The pH measurement was performed using a HI 221 potentiometer (Hanna Instruments, Chile), following a 1:10 (w/w) dilution in distilled water. The obtained result corresponds to the average of three determinations.^21^

##### 2.5.3.2 Surface Charge Determination

For the determination of surface charge (Zeta potential), the samples were diluted at a ratio of 5:1000 (w/w), using ultrapure water as the diluent, and analyzed at 25 °C. Dynamic light scattering (DLS) technology was employed via the electrophoretic mobility method using a Zetasizer Nano-ZS90 instrument (Malvern Instruments Ltd., Worcestershire, U.K.) with ultrapure water as the standard. The results were measured in mV, and determinations were performed in triplicate.^22^

##### 2.5.3.3 Creaming Index: Physical Stability

Physical stability was evaluated through visual analysis, as well as by measurements of the creaming index (CI) adapted from.^23^ First, a centrifugation test was performed, in which approximately 5 g (standardized to a total height of 3.5 cm) of each formulation was placed in test tubes and subjected to centrifugation in a Centribio model 80–2B centrifuge (Centribio, Brazil) at room temperature (25 °C), with a rotation speed of 3000 rpm for 30 min.^24^ The CI was calculated using eq 1

1 $$\mathrm{CI}\;\left(\%\right)=H_{s}/H_{t}\times 100$$

Where *H*_s_ and *H*_t_ represent the height of the serum layer (if destabilization occurred) and the fresh emulsion layer, respectively.

### 2.6 Rheological Characterization of the Optimized *Pickering* Emulsion

The rheological analysis of the *Pickering* emulsion containing andiroba oil was performed only on the stable formulation (F22) identified after the experimental design analysis. A Brookfield R/S Plus rheometer was used, equipped with a cone–plate geometry (C50–1/3 3183) and a Lauda RE206 thermostatic bath. The sample was previously homogenized to ensure uniformity and then subjected to rheological tests at three distinct temperatures: 20, 30, and 40 °C. The gap between the cone and plate was adjusted according to the manufacturer’s specifications.

Shear rate measurements were performed with progressively increasing rotational speeds, ranging from 50 to 1000 rpm in increments of 3 rpm, to generate the ascending curve. Then, the procedure was repeated in the reverse direction, with decreasing speeds from 1000 to 50 rpm, maintaining a variation interval of 3 rpm, to obtain the descending curve,^25^ with a total acquisition time of approximately 20 min for each curve (ascending and descending) per temperature. This protocol allowed for the evaluation of the rheological behavior of the emulsion, including the measurement of shear stress and viscosity. The data collected by the rheometer software were plotted in graphs of viscosity versus shear rate and flow curve using OriginLab software. The fitted rheological model was the Herschel–Bulkley (HB) model (User), with eq 2

2 $$\tau =\tau_{0}+K\cdot {\mathrm{\gamma ̇}}^{n}$$

Where τ is the shear stress (Pa), τ_0_ is the yield stress (Pa), *K* is the consistency index (Pa·s^*n*^), γ̇ is the shear rate (s^–1^), and *n* is the flow behavior index (dimensionless).

## 3 Results and Discussion

### 3.1 Physicochemical Characterization of Andiroba Oil

The characterization of andiroba oil was performed to understand its properties and ensure quality control for potential applications in topical formulations, as presented in Table 1.

**Table 1:** Physical-Chemical Characterization of Andiroba Oil (*C. guianensis* Aubl)

analyses	results
acid value	8.90 ± 0.13 mequiv KOH·g^–1^
peroxide value	4.56 ± 0.02 mequiv kg^1–^
iodine value	71g I_2_/100g
saponification value	194 mg KOH/g
relative density	0.906 ± 0.01
viscosity	76.65 ± 0.01 mm^2^/s
refractive index (25 °C)	1.46 ± 0.04
rancimat	5.20 h

The acid value of the andiroba oil was determined to be 8.90 ± 0.13 mequiv KOH/g^–1^, a value significantly higher than refined oil standards as reported in study^26^ (≤4.0 mequiv KOH/g^–1^). This indicates not only a significant presence of free fatty acids but also a possible advanced stage of hydrolytic degradation. As emphasized by,^26^ a high acid value compromises the oil’s oxidative stability and may limit its efficacy in topical formulations. However, it is important to note that this profile is common in unrefined crude oils, which according to^27^ retain free fatty acids with bioactive properties such as anti-inflammatory and wound-healing actions. Thus, while the high acidity may indicate degradation, it also reflects the oil’s unprocessed nature, which could be advantageous for therapeutic applications.

The peroxide value, measured at 4.56 ± 0.02 mequiv/kg, serves as an indicator of oil oxidation. According to,^28^ a peroxide value below 10 mequiv/kg is considered acceptable for vegetable oils. Thus, the value obtained for andiroba oil suggests good oxidative stability for safe and effective use in topical products. Oxidative stability may not only affect product appearance but also its therapeutic efficacy.^29^

The iodine value, measured at 71 g/100 g, indicates the amount of unsaturated fatty acids present in andiroba oil. Literature emphasizes that unsaturated fatty acids are highly valued in cosmetic formulations, as these compounds are known for their emollient and moisturizing properties, which help maintain skin barrier integrity and promote skin smoothness.^30^ Furthermore, unsaturated fatty acids play a crucial role in forming stable emulsions, particularly in *Pickering* emulsions that use solid particles as stabilizers. This suggests that andiroba oil is an appropriate choice for formulating emulsions aimed not only at enhancing hydration but also providing skin protection, thereby potentiating the benefits of cosmetic active ingredients.^31^

The saponification value, which was determined to be 194 mg KOH/g, indicates a significant content of fatty acids in the oil. This characteristic is favorable for emulsion formation, as a high saponification value is associated with greater emulsifying capacity.^32^ Emulsion stability is essential to ensure controlled release of active compounds, allowing the benefits of andiroba oil and other ingredients to be effectively delivered to the skin. The combination of a high iodine value and elevated saponification value reinforces the suitability of andiroba oil for cosmetic product applications, where efficacy and stability are paramount.^33^

The relative density of andiroba oil (0.90 ± 0.1) was close to that of water, a characteristic that favors the formation and stabilization of emulsions by minimizing phase separation through sedimentation, corroborating the findings of.^34^ The kinematic viscosity value (76.65 mm^2^/s) falls within a critical range for the performance of the final product. According to the literature, this viscosity is suitable for ensuring the rheological stability of the emulsion^35^ and positively influences sensory properties, such as skin adhesion and active release, without compromising spreadability.^36^ Together, these physicochemical properties indicate that the oil is a suitable ingredient for emulsified topical formulations.

The refractive index (RI) is an optical property representing the ratio between the speed of light in a vacuum and its speed in each medium. This parameter is influenced by variables such as temperature and chemical composition, with vegetable oils showing a direct correlation with the degree of unsaturation in the fatty acids present in triglycerides.^37^ Refractive index determination is crucial for quality control, particularly in processes involving thermal variations.^38^ In the present study, the andiroba oil’s refractive index was measured at 1.4605 ± 0.045, a value consistent with typical ranges reported in literature for vegetable oils, such as in studies by^39,40^ where andiroba oil showed indices ranging between 1.4594 ± 0.0001 and 1.4649 ± 0.0005. Furthermore, study^41^ mentions that refractive index is one of the parameters used in detecting olive oil adulteration, though emphasizes that RI alone is insufficient to identify adulteration, requiring combination with other analytical techniques such as FT-IR and GC-MS.

The Rancimat test is associated with oxidation, being one of the main factors affecting vegetable oil quality, leading to degradation of beneficial properties and formation of undesirable compounds.^42^ In this study, the andiroba oil showed an oxidation induction time of 5.20 h. This result indicates moderate oxidation resistance, a crucial factor for maintaining the oil’s quality and efficacy in formulations.^42^

The induction time of 5.20 h suggests that andiroba oil has acceptable, though not exceptional, stability. According to previous studies such as,^43^ oils with induction times exceeding 6 h are considered more stable, while those below this value may require additional handling and storage precautions. The high oleic acid content in andiroba oil may contribute to its oxidative stability, as this fatty acid is less susceptible to oxidation compared to polyunsaturated acids like linoleic and linolenic acids. Furthermore, the significant presence of saturated fatty acids such as palmitic acid also helps enhance the oil’s stability, since these acids tend to be more stable under oxidative conditions.^44^ Thus, the combination of these components may help improve andiroba oil’s resistance to oxidative degradation.

### 3.2 Determination of Fatty Acid Profile of Andiroba Oil by Gas Chromatography coupled to Mass Spectrometry (GC-MS)

The results obtained by gas chromatography coupled to mass spectrometry (GC-MS) of andiroba oil revealed the presence of major compounds representing four primary constituents, identified through the analysis, accounting for 99.99% of the oil’s components, of which 44.74% were saturated and 55.25% unsaturated. The chromatogram presented in Figure 1 demonstrates these principal constituents, with peak areas and heights detailed in Table 2.

**Figure 1:**
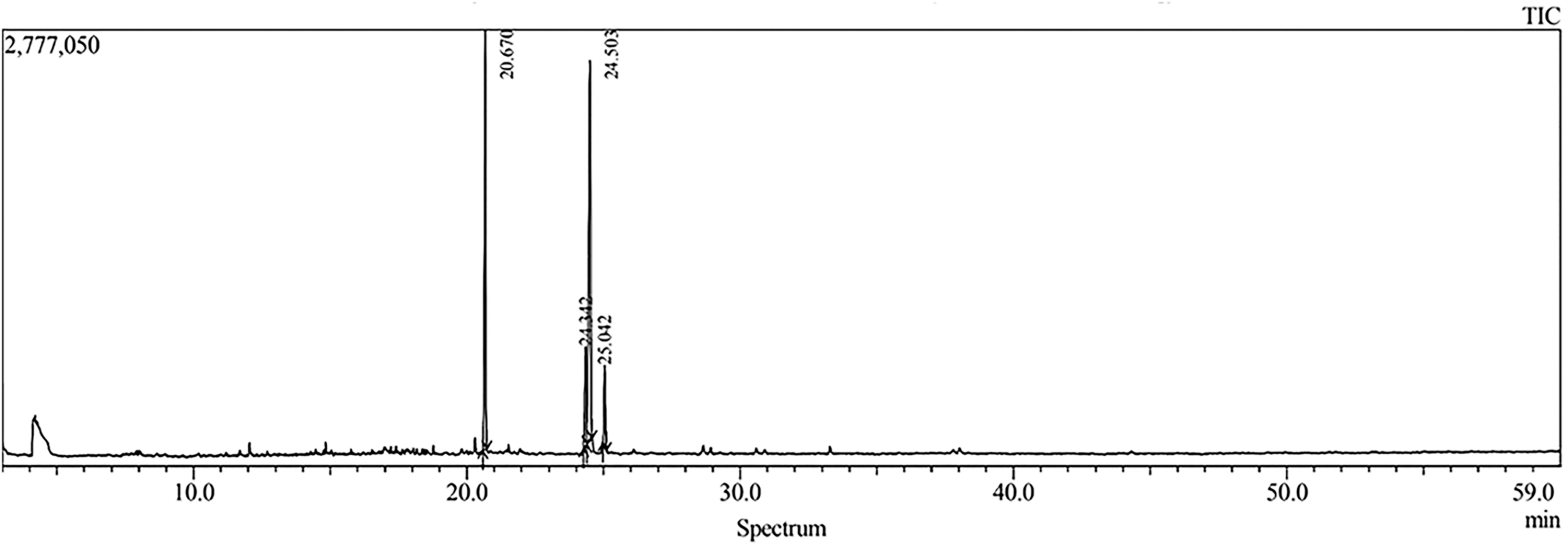
Chromatogram of fatty acid methyl esters from andiroba oil (*C. guianensis* Aubl) obtained by GC-MS.

**Table 2:** Fatty Acids Identified in Andiroba Oil by GC-MS

peaks	retention time	fatty acids	results (%)
1	20.670	oleic oil (9-octadecenoic acid)	44.68
2	24.342	palmitic (hexadecanoic acid)	37.30
3	24.503	stearic (methyl stearate)	7.44
4	25.110	linolenic (9,12-octadecadienoic acid (*Z*,*Z*))	10.57

Among the identified acids, palmitic acid and stearic acid are saturated fatty acids, characterized by the absence of double bonds in their carbon chains, while oleic acid and linolenic acid are unsaturated fatty acids, featuring one or more double bonds in their structures.^45^ Saturated fatty acids, such as palmitic and stearic acids, are known for their stability and resistance to oxidation, making them ideal for formulating products requiring an extended shelf life. They also form a protective barrier on the skin, enabling topical application by preventing water loss and facilitating the absorption of other bioactive compounds present in the oil.^46^ On the other hand, unsaturated fatty acids, such as oleic and linolenic acids, exhibit anti-inflammatory properties and contribute to hydration and emollience in cosmetic applications.^47^

Furthermore, the analysis of the peak areas indicates that 9-octadecenoic acid is the most abundant component of the oil, accounting for 44.68% of the total composition. This predominance suggests that andiroba oil may be a rich source of polyunsaturated fatty acids, which is advantageous for skin applications due to its role in regulating inflammation and melanin production.^48^ The results also show that methyl stearate, although present in a smaller amount (7.44%), may contribute to the emulsifying properties of the oil, making it an interesting option for cosmetic formulations.^49^

### 3.3 Andiroba Oil Profile and Fourier Transform Infrared Spectroscopy (FT-IR) Analysis

Fourier-transform infrared spectroscopy (FT-IR) was used to chemically characterize andiroba oil and Veegum. The obtained spectra allowed for the identification of the main functional groups present in each raw material,^50^ as illustrated in Table 3 for andiroba oil (Figure 2(a)) and Veegum (Figure 2(b)), respectively.

**Figure 2:**
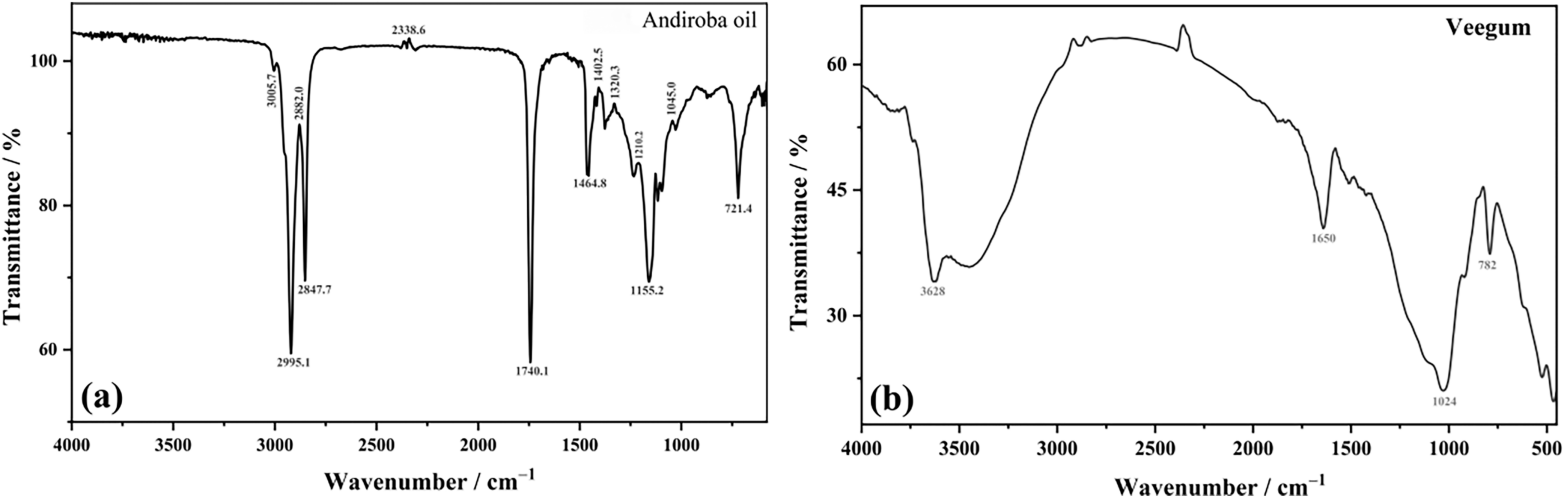
Infrared spectroscopic profile (FT-IR) of Andiroba Oil (a) and Veegum (b).

**Table 3:** FT-IR Absorption Bands and Vibrational Assignments for Andiroba Oil and Veegum

andiroba oil	
absorption wavenumber (cm^–1^)	functional group/vibrational mode assignment
3005.69	=C–H (stretching, alkenes)
2925.1	C–H (asymmetric stretching, –CH_2_–)
2882.01	C–H (symmetric stretching, –CH_3_)
2847.69	C–H (symmetric stretching, –CH_2_–)
1740.08	C=O (stretching, triglyceride esters)
1464.77/1402.53	C–H (bending, –CH_2_–)
1320.33/1210.21/1155.15/1045.02	C–O (stretching, esters, ethers)
721.84	=C–H (out-of-plane bending, alkenes)

Veegum	
absorption wavenumber (cm^–1^)	functional group/vibrational mode assignment
3628	O–H (stretching, silanol Si–OH groups)
1650	H–O–H (bending, adsorbed water)
1024	Si–O–Si (stretching, siloxanes)
782	Si–O and Al–O (deformation, clay structure)

In the andiroba oil spectrum, characteristic bands of vegetable oils were observed. The band at 3005.69 cm^–1^ is attributed to the =C–H stretching of double bonds, common in unsaturated fatty acids. The bands at 2925.10 and 2847.68 cm^–1^ correspond to the asymmetric and symmetric stretching of −CH_2_–, respectively, while the band at 2882.01 cm^–1^ is associated with the stretching of −CH_3_, all typical of long aliphatic chains in lipids.^51^ The intense band at 1740.08 cm^–1^ confirms the presence of carbonyl (C=O) groups from esters, characteristic of triglycerides.^52^ The bending vibrations of −CH_2_– and −CH_3_ appear at 1464.77 and 1402.53 cm^–1^, respectively.^53^ The region between 1320.33 and 1045.02 cm^–1^ shows bands of C–O stretching from esters and ethers, common in natural oils.^54^ Finally, the band at 721.84 cm^–1^ is associated with out-of-plane =C–H bending, reinforcing the presence of unsaturations.^55^ This spectrum is consistent with the fatty acid composition previously identified by GC-MS.

For Veegum, the results are consistent with its composition based on aluminum magnesium silicate. The broad band around 3628 cm^–1^ is characteristic of O–H stretching from silanol groups (Si–OH) present on the clay surface.^56^ The band at 1650 cm^–1^ is due to the H–O–H bending of adsorbed water molecules, indicating the hygroscopic nature of the material.^57^ The intense band at 1024 cm^–1^ is attributed to the asymmetric stretching of Si–O–Si bonds, predominant in layered silicate structures.^58^ Finally, the band at 782 cm^–1^ is associated with deformation modes of Si–O and Al–O bonds, typical of the structural network of clays such as montmorillonite.^59^ The FT-IR analysis confirmed the chemical identity of both components, validating their purity and suitability for the development of *Pickering* emulsions. The presence of hydrophilic groups (Si–OH) in Veegum is particularly relevant, as it contributes to the interfacial stabilization of the emulsion through adsorption at the oil and water phases.^60^

### 3.4 Thermal Behavior by Thermogravimetry (TG/DTG)

Thermogravimetric analysis (TGA) of andiroba oil (Figure 3a) revealed a main degradation event with an onset temperature of 357.33 °C and an endset temperature of 457.81 °C, resulting in a mass loss of 67.698% within this range. The high onset degradation temperature observed highlights the remarkable thermal stability of the oil.^61^ In this regard, it is evident that andiroba oil remains stable up to approximately 350 °C, which is consistent with its safe application as a raw material in industrial processes involving moderate heating steps. In contrast to these results, a previous study^62^ reported lower thermal stability for andiroba oil, with an onset degradation temperature of 234.5 °C. This discrepancy may be associated with variations in the material’s origin, extraction method, or degree of purity. The presence of a single, well-defined thermal event in the TGA curve suggests the homogeneity and purity of the analyzed material, indicating the absence of contaminants or other substances that could exhibit different thermal behaviors.^63^

**Figure 3:**
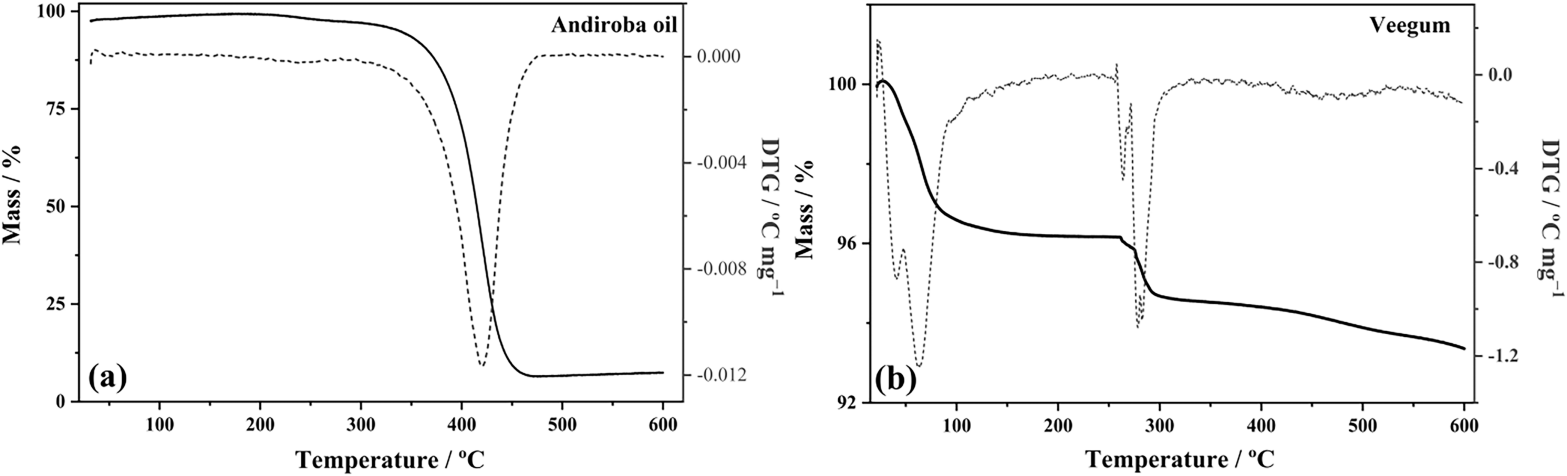
Thermogravimetric (TG) and derivative thermogravimetric (DTG) behavior of andiroba oil (a) and Veegum (b).

The degradation of andiroba oil can be attributed to the presence of fatty acids and other bioactive components, such as terpenes and flavonoids, which are known for their susceptibility to thermal degradation.^64^ Studies indicate that the chemical composition of andiroba oil, rich in unsaturated fatty acids, may contribute to its thermal reactivity, resulting in significant mass loss at elevated temperatures.^65^ The high mass loss observed suggests that, at temperatures above 357 °C, the oil’s components may degrade, potentially reducing its therapeutic efficacy and beneficial properties in emulsion formulations.^66^

Literature suggests that the thermal stability of vegetable oils can be improved by adding stabilizers, such as Veegum, which can help preserve the oil’s properties during storage and application.^67^ In this context, the derivative thermogravimetric (DTG) curve of andiroba oil, which shows a degradation peak at 420.47 °C, reinforces the idea that the oil’s thermal stability is a critical factor to consider in its use in formulations.

On the other hand, the thermogravimetric analysis (TG) of Veegum revealed two mass loss events, as shown in Figure 3(b). The first event, starting at 26.10 °C and ending at 69.81 °C, resulted in a mass loss of 1.2%. This phenomenon can be attributed to the removal of adsorbed moisture and dehydration of the silicate, a behavior commonly observed in hygroscopic materials. The presence of water in excipients can significantly influence their physical and chemical properties, affecting the stability and efficacy of topical formulations.^68^

The second mass loss event (6.50%), observed between 231.75 and 285.54 °C, is characteristic of the dehydroxylation of the clay structure. This process involves the removal of hydroxyl (OH̅) groups chemically bonded to the magnesium silicate network (laponite) that constitutes Veegum. The release of these structural water molecules in the form of vapor is a well-documented endothermic phenomenon for clays of this type and represents a significant alteration in their chemical structure.^69,70^

### 3.5 X-ray Diffraction (XRD) of Veegum

X-ray diffractometry was used to investigate the behavior of Veegum. Since its discovery, the importance of XRD has become immediately evident, establishing itself as a fundamental method for determining the atomic and molecular structures of various materials an essential prerequisite for understanding their properties.^71^ XRD is one of the main techniques for the microstructural characterization of crystalline materials and has recently been successfully applied to investigate a variety of structures and X-ray optical elements.^71^

It is a nondestructive method that provides phase-selective information on the state of residual stress in the surface region of (poly)­crystalline materials.^72^ The diffractogram of Veegum exhibited characteristic peaks at angular positions (2θ) of approximately 19.45; 21.68; 25.27; 27.83; 36.01; and 61.9°, as illustrated in Figure 4.

**Figure 4:**
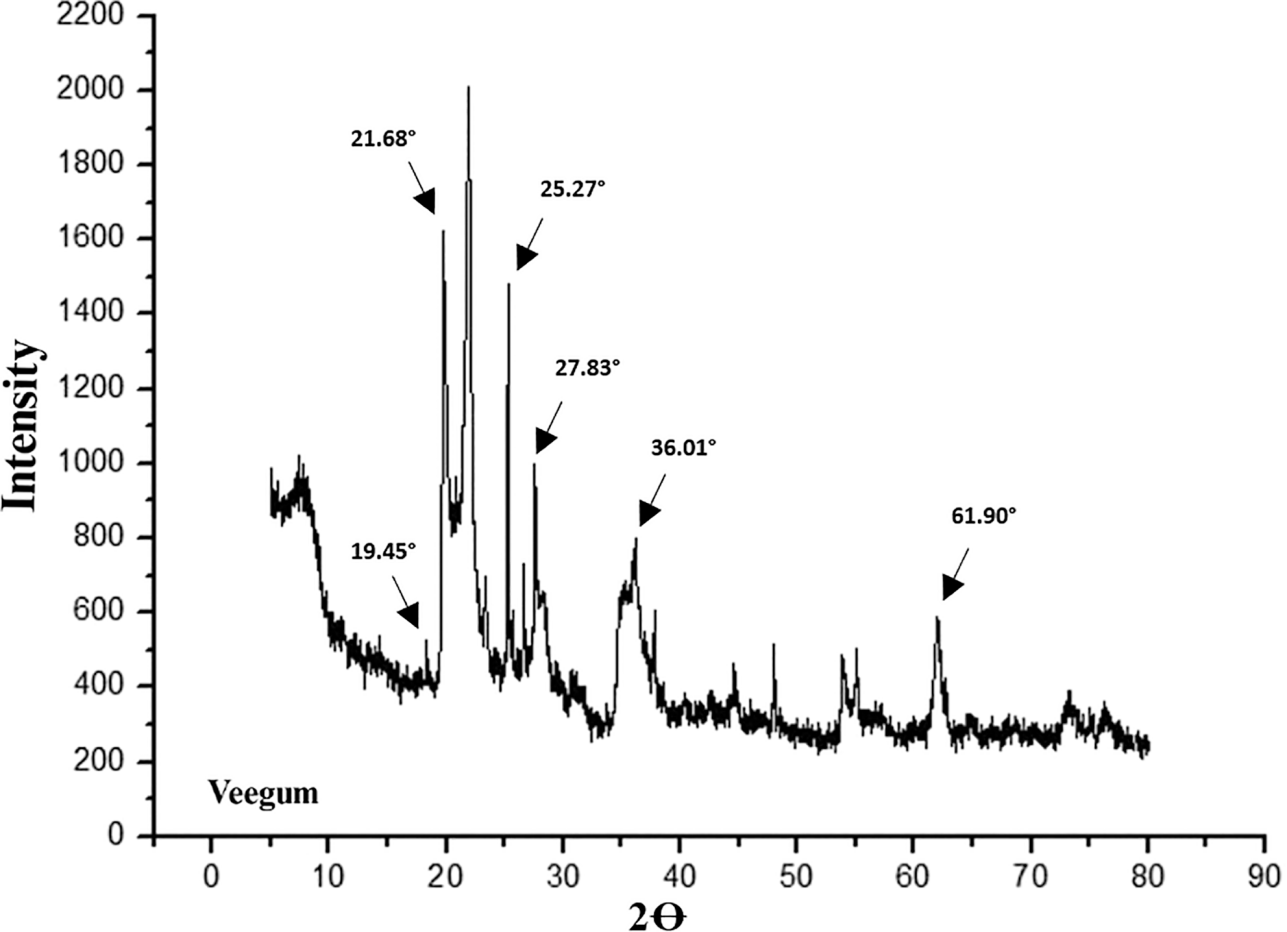
X-ray diffraction (XRD) of Veegum.

In a related study,^73^ used XRD to evaluate the influence of an aluminum magnesium silicate complex with gentamicin for drug delivery systems. The authors observed that the reflection peaks corresponding to the hybrid clay materials showed an increase in intensity, along with the emergence of additional peaks in the XRD patterns at 11.64, 18.91, and 20.90°.

These results confirmed the efficiency of the intercalation process and indicated the potential for introducing gentamicin into the interlayer space of montmorillonite. Thus, the resulting material demonstrated potential for use as a vehicle in modulated drug delivery systems. The diffractogram of Veegum, presented in the figure as mentioned above, exhibits peaks at similar positions (2θ ∼ 19.45; 21.68; 25.27; 27.83; 36.01; and 61.9°). Such evidence may suggest the formation of intercalated nanocomposites in Veegum, indicating that the material interacts electrostatically, leading to the formation of nanocomposite films.

### 3.6 Zeta Potential, Average Particle Size (Z-Average), and Polydispersity Index (PDI) of Veegum

The Zeta potential of Veegum was determined to be −38 mV ± 0.02. This significant negative surface charge is a key characteristic of aluminum magnesium silicate clays and is primarily attributed to the ionization of silanol groups (Si–OH) on the particle surface, which dissociate to form negatively charged silanolate (Si–O̅) groups in aqueous dispersion.^74^ A zeta potential value of −38 mV ± 0.02 suggests a moderate to good electrostatic stabilization potential. According to the literature, particles with zeta potential values greater than |±30| mV are generally considered to form stable dispersions due to strong electrostatic repulsion that prevents aggregation.^75^ This inherent negative charge is fundamental for the adsorption of Veegum particles at the oil–water interface, promoting the formation of a mechanical and electrostatic barrier that enhances emulsion stability.

The particle size analysis, expressed as the Z-Average, yielded a value of 1299 nm ± 0.04 (1.299 μm). This size indicates that the Veegum particles, as supplied, exist in a highly aggregated state. This aggregation is typical for hydrophilic clay minerals like Veegum (a purified smectite) in aqueous media, where face-to-face and edge-to-face interactions lead to the formation of larger tactoids or aggregates rather than fully exfoliated individual platelets.^76^ While this aggregate size is larger than the nanoscale often reported for primary clay platelets (which can be <100 nm), it is still highly effective for stabilizing *Pickering* emulsions. The literature shows that particles in the micrometer range can effectively stabilize emulsions by forming a dense, rigid layer around oil droplets, providing steric hindrance against coalescence.^77,78^

The Polydispersity Index (PDI) value of 0.672 ± 0.3 provides insight into the breadth of the particle size distribution. A PDI value close to 0.7 indicates a broad and heterogeneous size distribution.^79^ This is expected for a natural clay material that has undergone purification but not intensive size fractionation. A polydisperse system can be beneficial in emulsion stabilization, as a mixture of different particle sizes may allow for more efficient packing and coverage at the oil–water interface, potentially leading to a more robust interfacial film.^80^ However, a very high PDI can sometimes be associated with challenges in achieving perfectly reproducible dispersions. Despite the high PDI and aggregate size, the combination of a sufficiently negative zeta potential and the plate-like morphology of Veegum allows it to effectively stabilize *Pickering* emulsions, as demonstrated by the excellent creaming index and physical stability results obtained in this study.

### 3.7 Preparation of *Pickering* Emulsions

#### 3.7.1 Experimental Design: Central Composite Design for Evaluation of Emulsion Stability Processes

In recent years, one of the most notable uses of clays has been as a promising raw material for creating more stable systems for topical formulations, such as *Pickering* emulsions. In this context, the physicochemical properties of the emulsions, along with the concentration of excipients in the formulation, are crucial factors that influence the system’s ability to retain molecules and impact the long-term stability of *Pickering* emulsions.^81^ The 19 emulsions obtained from different stirring speeds and varying percentages of clay and oil (independent variables: *X*) were defined based on the matrix of the Rotational Central Composite Design (RCCD) study. These formulations underwent analyses of pH, Zeta potential, and creaming index (response variables: *Y*), in addition to a supplementary centrifugation analysis.

Table 4 provides a summary of the results obtained through the RCCD, conducted using a full factorial 2^3^ design with 5 replicates at the center point, employing *Statistica 8*.*0 software*. Three independent variables were used, enabling a total of 19 experiments.

**Table 4:** Matrix of the Rotatable Central Composite Design (RCCD) with Independent Variables and Responses of the Pickering Emulsions

	coded variables			uncoded variables			dependent variables		
runs	Veegum (%)	oil (%)	RPM	Veegum (%)	oil (%)	RPM	zeta potential (mV)	pH	creaming index (%)
1	–1	–1	–1	5	5	6000	–49.2 ± 0.85	6.58 ± 0.1	57.14
2	–1	–1	1	5	5	12,000	–41.7 ± 0.43	6.98 ± 0.3	42.85
3	–1	1	–1	5	10	6000	–41.5 ± 1.25	6.97 ± 0.1	28.57
4	–1	1	1	5	10	12,000	–43.26 ± 0.40	6.88 ± 0.2	28.57
5	1	–1	–1	10	5	6000	–41.36 ± 0.68	7.29 ± 0.6	20
6	1	–1	1	10	5	12,000	–36.9 ± 0.78	7.37 ± 0.4	28.57
7	1	1	–1	10	10	6000	–42.1 ± 0.62	7.33 ± 0.3	25.71
8	1	1	1	10	10	12,000	–39.23 ± 0.92	7.69 ± 0.1	22.85
9	–1.68	0	0	3.2	7.5	9000	–33.8 ± 0.98	7.27 ± 0.2	45.71
10	1.68	0	0	11.7	7.5	9000	–34.56 ± 1.87	7.2 ± 0.4	14.28
11	0	–1.68	0	7.5	3.2	9000	–35.13 ± 0.86	7.19 ± 0.5	42.85
12	0	1.68	0	7.5	11.7	9000	–35.13 ± 2.44	7.33 ± 0.2	25.71
13	0	0	–1.68	7.5	7.5	3954	–23.06 ± 1.27	7.34 ± 0.1	28.57
14	0	0	1.68	7.5	7.5	14,045	–37.3 ± 1.27	7.45 ± 0.7	31.42
15	0	0	0	7.5	7.5	9000	–32.46 ± 1.1	7.33 ± 0.6	31.42
16	0	0	0	7.5	7.5	9000	–33.4 ± 1.25	7.21 ± 0.8	31.42
17	0	0	0	7.5	7.5	9000	–36.03 ± 1.55	7.3 ± 0.2	31.42
18	0	0	0	7.5	7.5	9000	–34.3 ± 0.72	7.37 ± 0.1	31.42
19	0	0	0	7.5	7.5	9000	–35.86 ± 2.39	7.37 ± 0.2	34.28

In the analysis performed, it was found that trial 1 resulted in the formulation with the lowest pH of 6.58 ± 0.1, while trial 8 yielded the highest pH of 7.69 ± 0.1, both considered suitable for topical formulations. The formulations from the RCCD (Table 4) exhibited pH values between 6.58 ± 0.1 and 7.69 ± 0.1, while the Zeta potential ranged from −23.06 ± 1.27 to −49.2 ± 0.85. This variation is directly related to the amphiphilic nature of Veegum, whose surface charge is pH-sensitive.^82^ Formulations with lower pH (6.58 ± 0.1–6.98 ± 0.3) exhibited a more negative charge, as in trial 1, with −49.2 ± 0.85. This occurs because, at pH close to neutrality, the silanol groups (Si–OH) of Veegum undergo deprotonation (Si–O^–^), increasing the negative surface charge.^83^ Formulations with a less negative charge, such as trial 13 with −23.06 ± 1.27, though the only emulsion outside the stable range (>−30 mV) may have this reduction in charge magnitude associated with the partial neutralization of Si–O^–^ groups in an alkaline medium, as observed by^84^ in similar clays. Corroborating the study by,^85^ emulsions with a surface charge below −30 mV are classified as stable; however, in *Pickering* emulsions, other factors must be considered, such as the stirring speed during the emulsification process, as low speed can lead to the formation of emulsions with coalescence and flocculation, in addition to the need for an optimal concentration of solid particles.^86^ On the other hand, emulsions with a creaming index closest to zero are classified as phase separation-free.^87,88^ *Pickering* emulsion 10 came closest to this, with a creaming index of 14.28%, while the least favorable results were observed in trials 1, 2, 9, and 11.

#### 3.7.2 Evaluation of the Effects on the Regression of the Second-Order Polynomial Model

Figure 5 presents the Pareto diagrams for the response variables: (a) Zeta potential, (b) pH, and (c) creaming index. These graphs allow visualization of the magnitude and significance of the linear (*L*) and quadratic (*Q*) effects of the independent variables (Veegum concentration, andiroba oil concentration, and stirring speed) on each response. Values that exceed the significance line (*p* < 0.05) indicate statistically relevant effects. For example, it is observed that the creaming index was strongly influenced by the concentration of Veegum and andiroba oil, while Zeta potential and pH did not show significant effects under the tested conditions.

**Figure 5:**
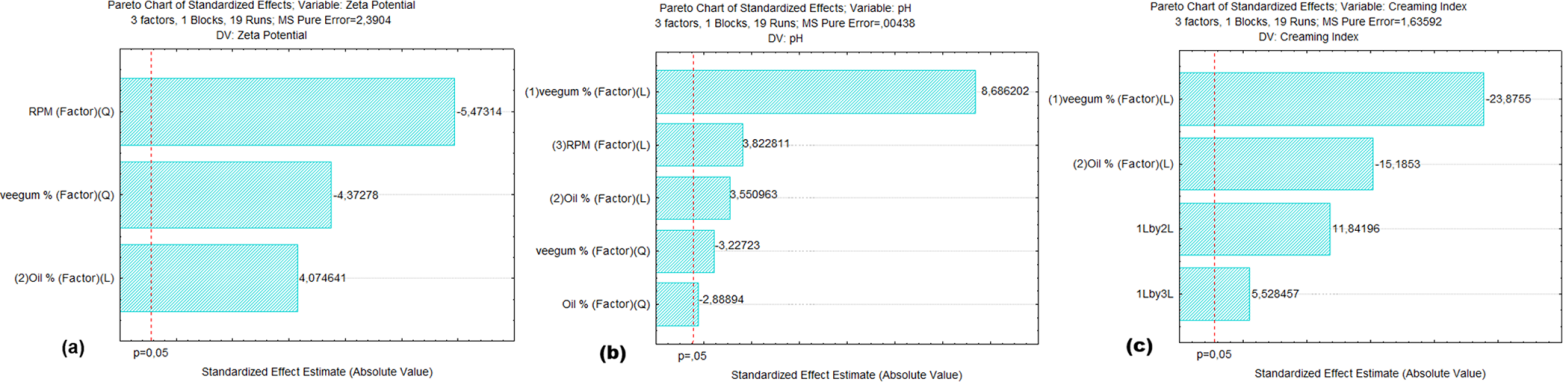
Pareto chart: analysis of the effects of variables on the Pickering emulsion. (*L*) linear effects and (*Q*) quadratic effects on (a) Zeta potential, (b) creaming index, and (c) pH.

The model for Zeta potential was not statistically significant (*p* = 0.753), with *R* ^2^ = 0.3511 and adjusted *R* ^2^ = 0.2016, indicating limited explanation of data variability. The Pareto chart (Figure 5a) revealed that, as dominant effects, only the quadratic effects of stirring speed (RPM) and Veegum concentration showed moderate magnitudes (absolute values of −5.47 and −4.37, respectively) but did not exceed the significance threshold (*p* > 0.05). Although Zeta potential was not significant, the obtained values (ranging from −23.06 to −49.2 mV) fell within the range considered stable for emulsions (>|30 mV|), supporting the efficacy of Veegum as a stabilizer^89^ and aligning with observations from^90^ on the relationship between electrostatic charge and resistance to coalescence.

The model for pH was also not significant (*p* = 0.132), with *R* ^2^ = 0.5145 and adjusted *R* ^2^ = 0.3769. The Pareto chart (Figure 5b) highlighted some relevant effects, with the quadratic effect of Veegum concentration (*p* ≈ 0.05) being the only one with marginal influence, reflecting a possible nonlinear relationship between pH and clay content. Furthermore, the pH of the formulations ranged from 6.58 to 7.69, within the ideal range for topical products (5.5–7.5).^91^ The literature emphasizes that Veegum tends to buffer the pH close to neutrality due to the presence of silanol groups (Si–OH), explaining the low variability observed.^92^

The model for the creaming index was highly significant (*p* < 0.0001), with *R* ^2^ = 0.9344 and adjusted *R* ^2^ = 0.9016, demonstrating excellent predictive capability. The Pareto chart (Figure 5c) revealed linear effects of Veegum (L) with a negative effect (standardized value = 23.88; *p* < 0.0001), indicating that higher concentrations (up to 11.7%) reduced phase separation. This occurs because solid particles form a colloidal network that prevents droplet coalescence.^93^ Additionally, Oil (L) had a positive effect (15.19; *p* = 0.002), showing that higher levels (>7.5%) increased the creaming index (57.14% in emulsion 1). This is due to an imbalance in the oil/water ratio, which saturates the particles’ adsorption capacity.^94^ On the other hand, the Veegum × Oil interaction showed a significant effect (11.84; *p* = 0.012), revealing that combinations with high Veegum and low oil minimized creaming. Finally, the stirring speed was not significant (*p* > 0.05), supporting studies stating that stirring energy has a limited impact after initial homogenization.^95^

#### 3.7.3 Evaluation of Reparameterized Models by ANOVA

To understand the significance of the results, analysis of variance (ANOVA) was applied to the responses from the 19 experiments. The *p*-value was used to evaluate the statistical significance of the model.

The analysis of variance (ANOVA, Table 5) revealed that the fitted model for the creaming index exhibited an F-value significantly higher than the tabulated value, with *p* < 0.0001, indicating strong statistical evidence that the model explains a large portion of the observed variance (93.44%, *R* ^2^ = 0.9344). However, significance was also observed in the lack of fit (*p* = 0.029), suggesting that the model may not fully capture all nuances of the experimental behavior or that variables not included in the model (e.g., room temperature, batch characteristics of the raw material) influenced the results.

**Table 5:** Analysis of Variance (ANOVA) for the Reparameterized Models of Zeta Potential, pH, and Creaming Index^a^

zeta	SQ	GL	MSQ	*F* te	*p* value
regression	65.812	4	16.4528907	0.47503201	0.753
residue	484.895	14	34.635331		
lack of fit	475.333	10	47.5333034	19.8850834	0.005
pure error	9.562	4	2.3904		
total SS	550.706				

pH	SQ	GL	MSQ	*F* teste	*p* value
regression	0.396	4	0.9888777	2.11973614	0.132
residue	0.653	14	0.04665098		
lack of fit	0.636	10	0.06355938	14.511273	
pure error	0.018	4	0.00438		
total SS	1.049	18			

IC	SQ	GL	MSQ	*F* teste	*p* value
regression	1589.177	4	397.294186	39.842352	*p* < 0.0001
residue	139.603	14	9.97165494		
lack of fit	133.059	1	13.3059489	8.13361834	0.029
pure error	6.544	4	1.63592		
total SS	1728.780				

a SQ: Sum of Squares (measures data variability); GL: Degrees of Freedom (number of independent values in the calculation); MSQ: Mean Square (SQ/GL; estimates variance); *F* te: *F* value (ratio between variances; tests model significance); *p* value: Probability (values <0.05 indicate significant effects) and Lack of fit: Tests whether the model fits the data well (*p* < 0.05 suggests inadequacy).

Despite the lack of fit, the model was retained for optimization purposes, given its high adjusted *R* ^2^ value and the consistency of the experimental data with the predictions.

On the other hand, Zeta potential (Y1) and pH (Y2) did not show statistical significance under the tested conditions (*p* > 0.05). For Zeta potential, the p-value was 0.753, and the model explained only 35.11% of the variation (*R* ^2^ = 0.3511). For pH, the p-value was 0.999, and the model showed low explanatory power (*R* ^2^ = 0.5145), indicating that the pH variation is not explained by the independent variables within the evaluated boundary conditions.

These results highlight the importance of the creaming index as the most critical parameter for evaluating the stability of *Pickering* emulsions, while Zeta potential and pH, though relevant, were not determinant under the tested experimental conditions. The statistical approach employed allowed for the identification of optimal formulation conditions, culminating in emulsion 22, which exhibited a creaming index of 0%, a Zeta potential of −40.5 mV, and a pH of 6.32, suitable for topical use.

Based on the reparameterized second-order polynomial model for the creaming index approved by ANOVA, eq 3 below was derived and used to make predictions and determine the optimal conditions for achieving formulations with the lowest possible creaming index from the evaluated variables

3 creaming(%)=120.26−9.8×Veegum−8.51×Oil+0.86Veegumoil

#### 3.7.4 Optimization via Desirability Function

Figure 6 illustrates the prediction and desirability profiles for the independent variables. In the upper graph, the variation of the creaming index as a function of Veegum and oil concentrations is observed, with the optimal region highlighted. In the lower panel, the individual desirability curves show how each variable contributes to the overall stability of the emulsion. The global desirability function (*D*) reaches its maximum value (*D* = 1.0) under conditions that minimize the creaming index, validating the optimized formulations.

**Figure 6:**
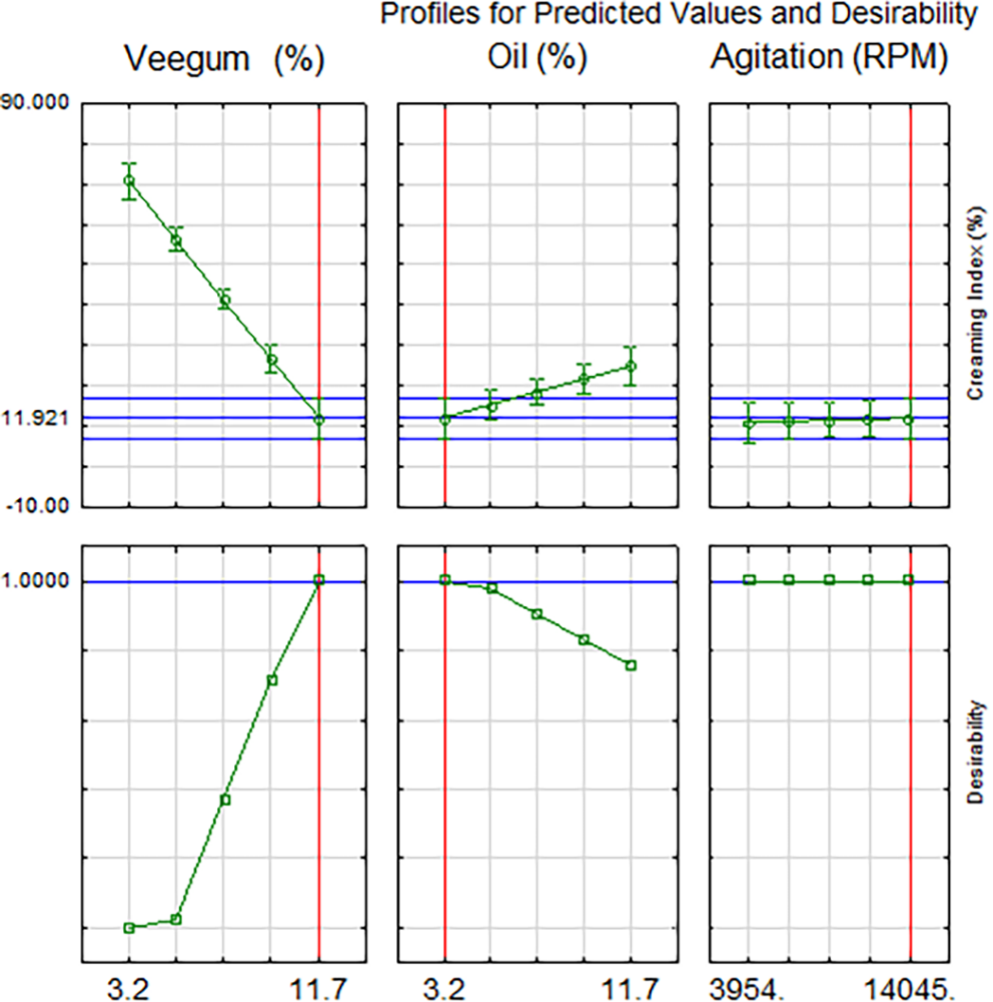
Predicted values and desirability plot.

The optimization revealed that the maximum desirability (*D* = 1.000) was achieved with the following parameters: Veegum: 11.7%; Andiroba oil: 3.2%; and stirring: 14,045 rpm. These values correspond to the optimal formulation with the predicted minimum creaming index (≈0%), as indicated by the descending curve in the right graph. It is observed that the individual desirability associated with the creaming index decreases substantially as this index increases, reinforcing its role as the main limiting factor for the physical stability of the emulsion.

In the lower panel, the individual desirability profiles indicate that the concentration of Veegum exerts a strong positive influence on desirability, with a significant gain between 5 and 11.7%. The oil content shows maximum desirability around 3.2%, with a progressive decline above this value, suggesting that higher concentrations may compromise the stability of the system. Additionally, it suggests that the stirring speed, although not statistically significant in the global model, maintained high desirability between 9,000 and 14,045 rpm, with stable behavior of the variable. The graphical approach facilitates the interpretation of interactions between factors and reinforces the applicability of the methodology in the rational formulation of *Pickering* emulsions.

The graph of predicted values and desirability (Figure 6) demonstrates that higher concentrations of Veegum (above 10% w/w) resulted in more negative values (close to −40 mV), indicating greater electrostatic stability. This behavior aligns with studies attributing the negative charge of aluminum magnesium silicate to the deprotonation of silanol groups (Si–OH) at neutral pH, forming Si–O^–^, which repel droplets and prevent coalescence.^96,97^ The interaction between Veegum and oil was also significant, corroborating the hypothesis that solid particles act as physical barriers, reducing interfacial tension between phases.^98^

Furthermore, it revealed an optimal point for oil concentration (between 3.2 and 5% w/w), beyond which stability decreased. This phenomenon can be explained by the increase in the oil-to-water phase ratio, which promotes flocculation and phase separation, as observed by^99^ in conventional emulsions. Moreover, excessive oil may saturate the adsorption capacity of Veegum particles, compromising their effectiveness as stabilizers.^100^

Although stirring speed (RPM) did not show statistical significance in the model (*p* > 0.05), the graph indicates that extremely low values may lead to less homogeneous emulsions. This suggests that once minimal energy for particle dispersion is achieved, additional variations have limited impact, consistent with studies prioritizing solid particle concentration as the determining factor.^101^

The selection of experimental conditions corresponding to formulations 20, 21, and 22 in Table 5 was based on the analysis of optimization and desirability profiles. The profile plot (Figure 6) showed that the highest desirability values were obtained in the upper range of Veegum concentration (11.7%) associated with low levels of andiroba oil (2.0 to 3.2%) and high stirring speed (14.045 rpm).

The region of the experimental space identified as ideal for minimizing the creaming index (the main response variable) proved compatible with maintaining pH and zeta potential within ranges suitable for stability and topical use. Individual desirability analysis indicated that Veegum concentration exerted an increasing positive influence, reaching a maximum value at 11.7% and remaining high at 14%, justifying the selection of formulations 20 (11.7%), 21 (11.7%), and 22 (14%), in line with the literature.^102^ Although the predictive model indicated a creaming index of 11.9% for formulation 20, the experimental result revealed a significantly lower value (5.71%), demonstrating a model underestimation. This discrepancy may be associated with nonlinear interactions or uncontrolled variables in the emulsification process. Given this, additional formulations (21 and 22) with Veegum contents maintained at 11.7 and 14%, respectively, were tested to validate the trend of improved physical stability and explore optimization potential beyond initially predicted. Formulation 22, in particular, exhibited a null creaming index (0%), confirming the efficacy of increasing Veegum concentration in stabilizing the emulsion and validating the adjustment strategy based on experimental response.

Additionally, the combination of these factors resulted in negative predicted values for the creaming index (indicating high stability) and global desirabilities close to 1, reinforcing the suitability of the selected formulations for experimental validation. Trial 22, in particular, was the point of maximum desirability, considered the optimal formulation due to its null creaming index (0%), zeta potential of −40.5 mV ± 0.8, and pH of 7.45 ± 0.72, compatible with dermatological applications.

Table 6 presents the results obtained for the optimized formulations in triplicate (20, 21, and 22), tested under the conditions predicted by the desirability function. The experimental parameters were compared with the values predicted by the model, particularly the creaming index, with an analysis of the confidence interval.

**Table 6:** Herschel–Bulkley Rheological Model Parameters Fitted for the Optimized *Pickering* Emulsion (Formulation 22) at Different Temperatures^a^

optimized emulsions	20	21	22
veegum (%)	11.7	11.7	14
oil (%)	3.2	2	3.2
unrest (RPM)	14,045	14,045	14,045
zeta (mV)	–43.1 ± 1.28^a^	–38 ± 1.44^a^	–40.5 ± 0.80
pH	7.45 ± 0.60^a^	7.45 ± 0.09^b^	7.45 ± 0.72^c^
creaming index (%)	5.71 ± 1.65^a^	8.57 ± 1.64^b^	0%^c^
predicted creaming index (%)	11.9 ± 6.1	10.11 ± 6.91	–4.01 ± 7.88

a *Means with different letters in the same row are different from each other according to Tukey’s test (*p* <0.05).

Formulation 22 validated the results of the predictive model and the optimization via the desirability function, being considered the most stable and suitable among all tested. The absence of critical divergence between the observed and predicted values confirms the adequacy of the mathematical model and justifies its use as a predictive tool for future formulations.

The application of Tukey’s test in the analysis of the optimized *Pickering* emulsions (Formulations 20, 21, and 22) allowed for a robust statistical comparison of the response variables (Zeta potential, pH, and creaming index), elucidating significant differences between the formulations. Tukey’s test (Table 6) confirmed statistically significant differences (*p* < 0.05) between the formulations, particularly in the creaming index, where Formulation 22 exhibited superior performance compared to the others. This reinforced the overall significance of the model (*p* < 0.0001), validating the experimental approach.

### 3.8 Rheological Characterization of Optimized *Pickering* Emulsion with Andiroba Oil

Rheological characterization is essential for understanding the behavior of complex systems such as *Pickering* emulsions, which are stabilized by solid particles and exhibit specific functional properties. In the present study, the *Pickering* emulsion containing andiroba oil (formulation 22, with no phase separation) demonstrated pseudoplastic flow behavior (Figure 7), characterized by a decrease in viscosity with increasing shear rate, constituting a non-Newtonian system typical of structured emulsions.^92^ Such behavior is desirable in topical formulations, as it allows the product to exhibit high viscosity at rest, ensuring stability, while becoming more fluid during application, facilitating spreadability.

**Figure 7:**
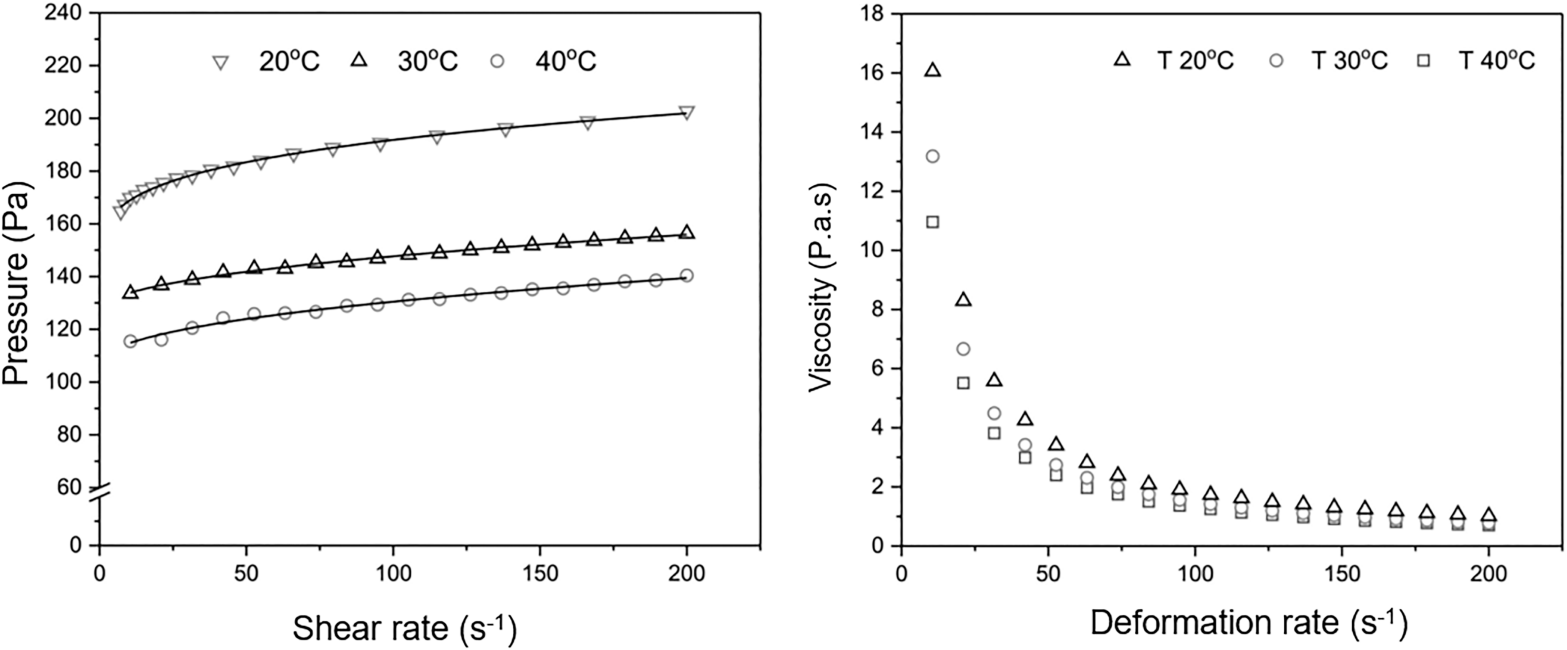
Viscosity behavior versus shear rate at temperatures of 20 °C, 30 and 40 °C (a) and Flow curve (shear stress × shear rate) (b).

Moreover, the emulsion exhibited high shear stress, attributed to the formation of a structural network promoted by Veegum, a thickening and stabilizing agent. The yield stress is a relevant parameter, as it indicates the minimum force required for the material to start flowing, providing consistency and resistance to unwanted shear.^103^ The absence of observed thixotropy suggests that the emulsion’s microstructure is stable under shear, which is advantageous for products subjected to varying stress during handling and application.^104^

The fit of the Herschel–Bulkley (HB) model, illustrated in Table 7, to the experimental data, with a coefficient of determination *R* ^2^ greater than 0.98, confirms that this mathematical model is suitable for describing the rheological behavior of the emulsion, characterizing complex systems that exhibit fluidity under shear stress.^105^ The parameter P3, which ranged between 0.255 and 0.47578, indicates sensitivity to shear while maintaining structural stability.

**Table 7:** Optimized Pickering Emulsion Formulations and Their Respective Experimental and Predicted Responses

ModelHB	20 °C	30 °C	40 °C
P1	139.6563 ± 5.30741	126.74528 ± 1.49128	105.94864 ± 3.4367
P2	16.11651 ± 4.04894	2.34349 ± 0.6132	3.10234 ± 1.52622
P3	0.255 ± 0.03218	0.47578 ± 0.04054	0.44916 ± 0.07496
R-Square (COD)	0.99672	0.99614	0.98666
reduced Chi-Sqr	0.4543	0.18349	0.79579
Adj. R-Square	0.99631	0.99566	0.98499

Analysis of the temperature effect revealed that parameter P1, related to apparent viscosity, decreased significantly as the temperature increased from 20 to 40 °C. This phenomenon is explained by greater molecular mobility and weakened interactions between particles in the emulsion, resulting in reduced flow resistance.^106^ Considering that the physiological temperature of the skin is approximately 32 °C, this variation is critical for dermocosmetic applications, as it may influence sensory perception and product efficacy.^107^ However, the small variation in parameter P3 between 30 and 40 °C indicates that the emulsion maintains its rheological stability under near-physiological conditions, which is desirable to ensure product performance during use.

The rheological characterization demonstrated robust and predictable pseudoplastic behavior, with high yield stress and thermal stability fundamental attributes to ensure both product stability and sensory performance. Modeling using the Herschel–Bulkley fit (*R* ^2^ > 0.98) reinforces the formulation’s ability to behave as a reversible thixotropic system, suitable for topical applications with demanding aesthetic and functional requirements.

The findings of this research are fundamental for the development of future stages, such as the incorporation of one or more drugs, as well as the evaluation of the stability of the formulation, since response variables influence such processes. Formulation 22 from the study exhibited ideal behavior for topical cosmetics, combining excellent physical stability (creaming index = 0%), robust rheological profile, and a composition based on natural active ingredients (andiroba oil). The obtained data confirm the potential of Veegum as an effective particulate stabilizer, highlighting its ability to promote kinetic and structural stability in *Pickering* emulsions. The observed pseudoplasticity aligns with sensory requirements for topical products, ensuring drip resistance with high spreadability.

## 4 Conclusion

This study successfully demonstrated the development and optimization of innovative *Pickering* emulsions using andiroba oil (*C. guianensis* Aubl) and stabilized with aluminum magnesium silicate (Veegum). The approach employed overcame the instability limitations common to conventional emulsions, offering a robust and efficient alternative for topical applications. Detailed physicochemical characterization of andiroba oil confirmed its high quality and thermal stability, with a fatty acid profile rich in unsaturated compounds, corroborating its potential for cosmetic and pharmaceutical applications. The use of Rotational Central Composite Design (RCCD) proved to be a powerful tool for system optimization, enabling the identification of ideal formulation conditions that resulted in emulsions with excellent physicochemical stability. The optimized emulsion (Formulation 22) stood out for its exceptional stability, evidenced by a highly negative Zeta potential (−40.5 mV), complete absence of creaming (0%) after centrifugation, and a pH (7.45) within the ideal range for topical products. Rheological analysis revealed pseudoplastic behavior, perfectly fitted to the Herschel–Bulkley model, which provides the product with adequate viscosity to ensure high stability at rest and facilitated spreadability during application a highly desirable characteristic in dermatological formulations. The results not only validate Veegum as an extremely effective particulate stabilizer for *Pickering* emulsions but also position andiroba oil as a valuable natural asset for the cosmetic and pharmaceutical industries, promoting the sustainable valorization of Amazonian biodiversity. Based on the promising results, subsequent studies are necessary to explore the platform’s potential for controlled active release through in vitro permeation and release assays. Validation of the proposed therapeutic benefits, such as emollient and anti-inflammatory efficacy, should be the next crucial step through in vivo evaluation models. Furthermore, the system’s versatility encourages investigation into its ability to deliver other insoluble active principles or incorporate different vegetable oils from the Amazonian biodiversity, expanding the range of cosmeceutical and pharmaceutical applications. Thus, this study contributes to the valorization of natural resources and offers a promising technological platform, whose full potential should be unveiled through these future researches, directing it toward concrete innovations in the topical product industry.
